# RescQ™: Study protocol for a feasibility pilot of structured support for bystanders/lay-rescuers following out-of-hospital cardiac arrest

**DOI:** 10.1016/j.resplu.2026.101445

**Published:** 2026-08-08

**Authors:** Uzma Sajjad, Marco Mion, Rupert Simpson, Guilherme Movio, Sarosh Khan, Liz Sharpe, Jean Davis, Luke Roberts-Andreou, Gareth Grier, Paul Swindell, Stuart Menzies, Rob Major, Blake Powell, Michael Watts, Nilesh Pareek, Thomas R. Keeble

**Affiliations:** a Department of Cardiology, Essex Cardiothoracic Centre, Mid and South Essex NHS Foundation Trust, Basildon, UK; b Anglia Ruskin School of Medicine & MTRC, Chelmsford, UK; c King’s College Hospital, London, UK; d East of England Ambulance Service NHS Trust (EEAST), Cambridge, UK; e Essex and Herts Air Ambulance (EHAAT), UK; f Sudden Cardiac Arrest UK (SCAUK), Benfleet, UK; g East Anglian Air Ambulance (EAAA), Norwich, UK; h Blum Health Ltd, London, UK; i School of Cardiovascular and Metabolic Medicine & Sciences, British Heart Foundation Centre of Excellence, King’s College London, UK

**Keywords:** Bystanders in OHCA, Psychological first aid for bystanders, Post-traumatic stress symptoms in OHCA bystanders, Feasibility pilot for bystander support

## Abstract

Worldwide, 3.8 million out-of-hospital cardiac arrests (OHCA) occur annually. RescQ™ is a 12-month, non-randomised, prospective feasibility pilot evaluating a structured support pathway for adult bystanders involved in out-of-hospital cardiac arrest of presumed medical or cardiac aetiology across Essex, United Kingdom.

The pathway was developed to address the absence of a nationally standardised post-event support process for bystanders who witness, call emergency services, or assist with resuscitation. Attending ambulance clinicians offer brief on-scene signposting and register consenting adult bystanders through a dedicated digital application.

Registration triggers a single text message or email linking to the RescQ™ website, which provides information on common physical and psychological reactions after involvement in cardiac arrest, coping strategies, lived-experience videos and sources of further support. Bystanders can self-refer to a liaison officer for structured supportive contact, psychological screening (using Clinical Outcomes in Routine Evaluation and the National Stressful Events Survey Short Scale), event-related discussion, signposting and onward referral where required.

The primary outcome is feasibility, assessed through ambulance clinician uptake, bystander website access and acceptability to ambulance clinicians and bystanders. Secondary outcomes include digital engagement, psychological screening data, liaison officer activity, onward referral, satisfaction and service-delivery resource use.

The project was reviewed by the West of Scotland Regional Ethics Committee and classified as a service evaluation. At the time of revised submission, the 12-month pilot has been completed, and data analysis is underway.

## Introduction

Worldwide, there are 3.8 million out-of-hospital cardiac arrests (OHCA) recorded annually^1^ of which 115,000 are reported to the ambulance services in the UK and resuscitation is attempted in around 43,000.^2^ More than half of these events are witnessed and around 70% occur at home^2^, ^3^, ^4^ Bystanders to these events may be family members, friends, colleagues, or total strangers, with rate of bystander cardiopulmonary resuscitation (CPR) varying substantially between regions, from 13% to 82%.^5^ In England, national OHCA registry data reported bystander CPR in 72.6% of cases.^2^

The experience of witnessing, providing first aid or helping in an OHCA can be extremely distressing and may have lasting emotional and psychological consequences for those involved.^6^, ^7^, ^8^, ^9^ Bystanders who perform CPR or assist during an OHCA are thrust into an emotionally traumatic situation without warning, potentially leading to acute stress reactions, including hyper-alertness, intrusive memories, flashbacks, sleep disturbances, anxiety, guilt, and helplessness.^9^, ^10^, ^11^, ^12^ These emotional responses can persist for days to months, impacting the mental well-being of the bystander.^10^ Some are so traumatised that they are unable to carry on with normal day-to-day activities.^10^, ^11^, ^12^, ^13^

Post-traumatic stress symptoms (PTSS) are also common.^12^ Long-term effects such as anxiety, grief, and isolation can persist for years, with approximately 19% to 30% of bystanders experiencing high levels of distress even two years after the incident.^14^

In the time-pressured environment of an OHCA, the well-being of the bystander frequently becomes secondary, if a consideration at all.^11^, ^13^, ^15^ Family members present at the event, increasingly termed 'co-survivors' or 'key supporters', may receive some support from prehospital and hospital staff following patient admission.^16^, ^17^ However, if the patient dies at the scene, this hospital-based support is no longer available.^9^, ^11^, ^15^ Moreover, bystanders who are strangers to the patient typically do not receive any support, leaving them to cope with the emotional aftermath on their own.^18^

There is currently no standardised psychological support programme for OHCA bystanders in the UK. While some air ambulance charities and ambulance services offer aftercare following OHCA or other critical incidents, provision is inconsistent and there is no nationally standardised approach. A Norwegian study of first aid providers reported that, among those followed up beyond one month, 81% continued to report at least one symptom or functional impairment, and 19% met criteria for post-traumatic stress disorder or complex post-traumatic stress disorder.^19^

An exploratory survey at Essex Cardiothoracic Centre (ECTC) and among Sudden Cardiac Arrest UK (SCAUK) group members, assessed the psychological impact of witnessing or assisting in OHCA. Among 204 respondents, post-traumatic symptoms were prevalent across all groups, affecting 90% of untrained bystanders, 67% with prior CPR training, and 46% with advanced CPR training. Despite 74% expressing a strong need for immediate support, most received little or no formal debriefing. Advanced-trained individuals reported a more positive experience, possibly reflecting their higher rate of informal debriefing (22%).^6^

A recent scoping review identified emerging support interventions for OHCA bystanders, but found that existing provision was inconsistent, variably promoted and largely unevaluated. Few studies reported participant outcomes, and none evaluated intervention effectiveness.^20^

The RescQ™ pilot was conceived to address this critical gap. It aims to provide timely and practical psychological support for bystanders and lay-rescuers through an integrated digital platform and personalised follow-up. By embedding RescQ™ into prehospital care, the project seeks not only to support bystanders in the immediate aftermath of OHCA but also to generate evidence on feasibility, acceptability, and impact.

This manuscript describes the design and methodology of the RescQ™ feasibility pilot.

## Methods and analysis

### Design

RescQ™ pilot is a 12-month, non-randomised, prospective, observational study to support consecutive bystanders involved in OHCA of presumed cardiac/medical aetiology across Essex. This is a feasibility pilot integrating prehospital and hospital services, and patient focus groups.

### Ethics

The study was reviewed by the West of Scotland Regional Ethics Committee (REC Ref: 24/WS/0088; IRAS ID: 340729) and classified as a service evaluation, meaning formal National Health Service (NHS) REC and Health Research Authority (HRA) approval is not required. It is conducted in compliance with NHS research governance principles and General Data Protection Regulation (GDPR), with information governance and data protection frameworks established in collaboration with prehospital and hospital information teams.

### Eligibility (Table 1)

All adults (age ≥18 years), consenting bystanders who have witnessed and/or called 999 and/or directly assisted in resuscitation efforts at an OHCA of cardiac/medical aetiology. The bystanders should be willing to engage with the digital platform and/or follow-up surveys if needed.

**Table 1:** Eligibility criteria for RescQ™ (OHCA: out-of-hospital cardiac arrest; CPR: cardiopulmonary resuscitation).

**Inclusion criteria**
Consenting bystanders
Witnessed the OHCA, and/or called 999, and/or performed CPR, and/or assisted in resuscitation efforts
OHCA of presumed cardiac or medical aetiology
Willing to engage with the digital platform and/or follow-up surveys

Bystanders who cannot consent to participation or those involved in OHCA of non-cardiac/non-medical aetiology (traumatic, road traffic accidents, drug overdose, suicide, drowning) will be excluded.

### Rationale for eligibility

Bystanders who witness or assist during an OHCA are at high risk of psychological distress and are therefore the appropriate target population. Inclusion is restricted to adults (≥18 years) as the RescQ™ team works exclusively within adult services and lacks the expertise and safeguarding structures required to support a paediatric population, though bystanders under 18 are recorded for descriptive purposes.

The study is limited to cardiac/medical OHCAs, as traumatic or non-cardiac arrests involve different contexts, clinical management, and potentially more complex psychological sequelae, which fall outside the project team's expertise.

Bystanders unable to provide informed consent are excluded, as participation involves survey completion and potential LO engagement.

These criteria ensure the feasibility pilot targets those most likely to benefit, within the scope and resources of the participating teams.

### Stakeholder engagement and patient and public involvement

RescQ™ has been co-designed with input from cardiac arrest survivors, bystanders, psychologists, mental health counsellors, prehospital clinicians, and patient focus groups, who informed the website content, app design, and overall pathway structure.

The prehospital team includes representatives from the East of England Ambulance NHS Trust (EEAST), East and Herts Air Ambulance Trust (EHAAT), and East Anglia Air Ambulance (EAAA). The hospital team, based at ECTC comprises cardiologists, psychologists, occupational therapists, and mental health counsellors. The digital platform was developed in partnership with Blum Health Ltd.

Patient and public involvement is provided through the Sudden Cardiac Arrest UK (SCAUK) group, whose members contributed lived experience as survivors, co-survivors and bystanders. Contributors informed the design of the RescQ pathway, website content, tone of communication, acceptability of on-scene registration, and the need for flexible access to support.

### Geographical setting

In the UK, an OHCA typically begins when a bystander witnesses a collapse and calls 999. Call handlers guide CPR until professional help arrives. The study is being conducted in Essex (population 2.8 million), UK, within the regional OHCA response pathway served by EEAST land ambulance, and EHAAT and EAAA air ambulance services. Usually community first responders (CFRs) are often first on scene, followed by EEAST land ambulance clinician crews providing advanced life support, with air ambulance support from EHAAT or EAAA where needed.

If return of spontaneous circulation (ROSC) is achieved, patients are transferred to a local emergency department or the ECTC depending on clinical need. Family members may accompany the patient in the land ambulance or make their own way to hospital. In cases where resuscitation is unsuccessful, the patient’s body is taken to the local morgue.

While ambulance crews may offer a short debrief or leave a contact card, this is inconsistent and dependent on scene dynamics and crew workload. As a result, the psychological impact on bystanders is rarely addressed in a structured way, and those experiencing post-event distress are currently directed to primary care, with no dedicated follow-up pathway.

### Intervention (Fig. 1, Fig. 2)

#### Ambulance clinician registration

At the OHCA scene, attending ambulance clinicians provide a brief standardised debrief towards the end of resuscitation, thanking bystanders for their actions, acknowledging potential psychological impact, and offering support through the RescQ™ pilot.

Registration is completed via the RescQ™ app on EEAST iPad devices, which records the event with geo-location and timestamping and generates a unique quick response (QR) code. This can be scanned by other clinicians to register multiple bystanders, or by bystanders themselves to enter their own contact details (email or mobile number). The app also captures whether the bystander is known to the patient and records the number of bystanders under 18 or who decline registration.

Following registration, the bystander’s contact information is transferred immediately to a secure central server and automatically deleted from the ambulance clinician-facing application. Clinicians are therefore unable to access email addresses or mobile telephone numbers after the event has been registered. No additional personal information is stored within the application.

#### Digital prompt

Upon registration, bystanders receive a single text or email containing a brief introduction to the RescQ™ pilot and a secure link to the dedicated website, accessible at any time. No reminders or further unsolicited communications will be sent to respect privacy and avoid intrusion.

#### Website resources

The RescQ™ website is accessible only to registered bystanders and provides resources on the expected psychological and physical reactions to witnessing an OHCA or performing CPR, including practical advice, coping strategies, and guidance on seeking further help. Content includes psychologist-authored articles and selected open-access resources from SCAUK and the Resuscitation Council UK (RCUK).

The website also hosts 16 short videos featuring survivors, bystanders, cardiologists, psychologists, occupational therapists, and mental health counsellors, drawing on both lived experience and clinical expertise.

Bystanders requiring additional support can self-refer via a dedicated button, providing contact details and consent for follow-up from a liaison officer (LO), who will then reach out to offer further assessment and signposting.

Representative screenshots of the website homepage, resource library and liaison officer self-referral page are included in Fig. 3.

**Fig. 1:**
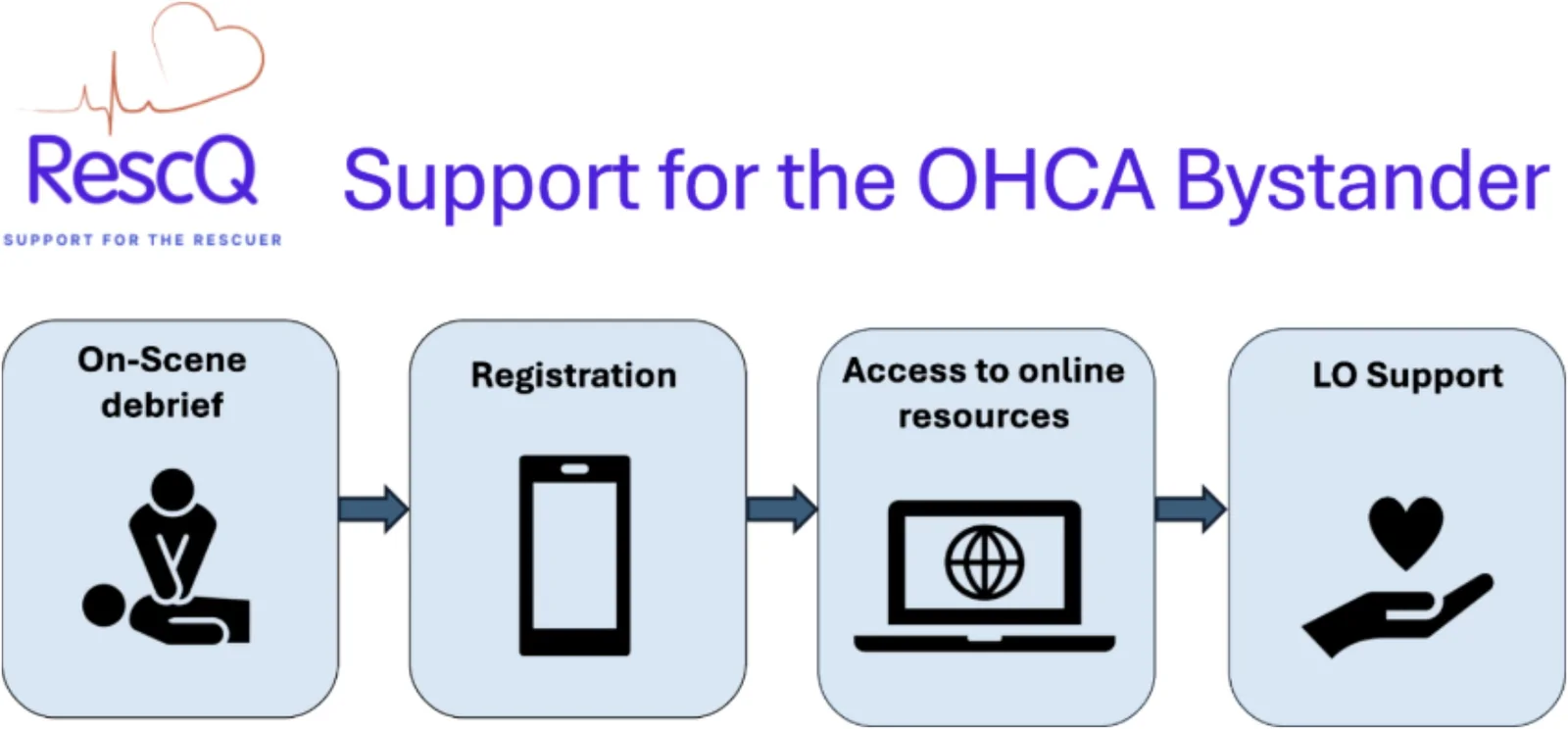
**Schematic overview of the RescQ™ pathway for bystanders involved in out-of-hospital cardiac arrest**. Following an on scene debrief, bystanders are offered registration to RescQ™, access to digital support resources, and escalation to liaison officer support where additional needs are identified. **OHCA:** out-of-hospital cardiac arrest; **LO:** liaison officer.

**Fig. 2:**
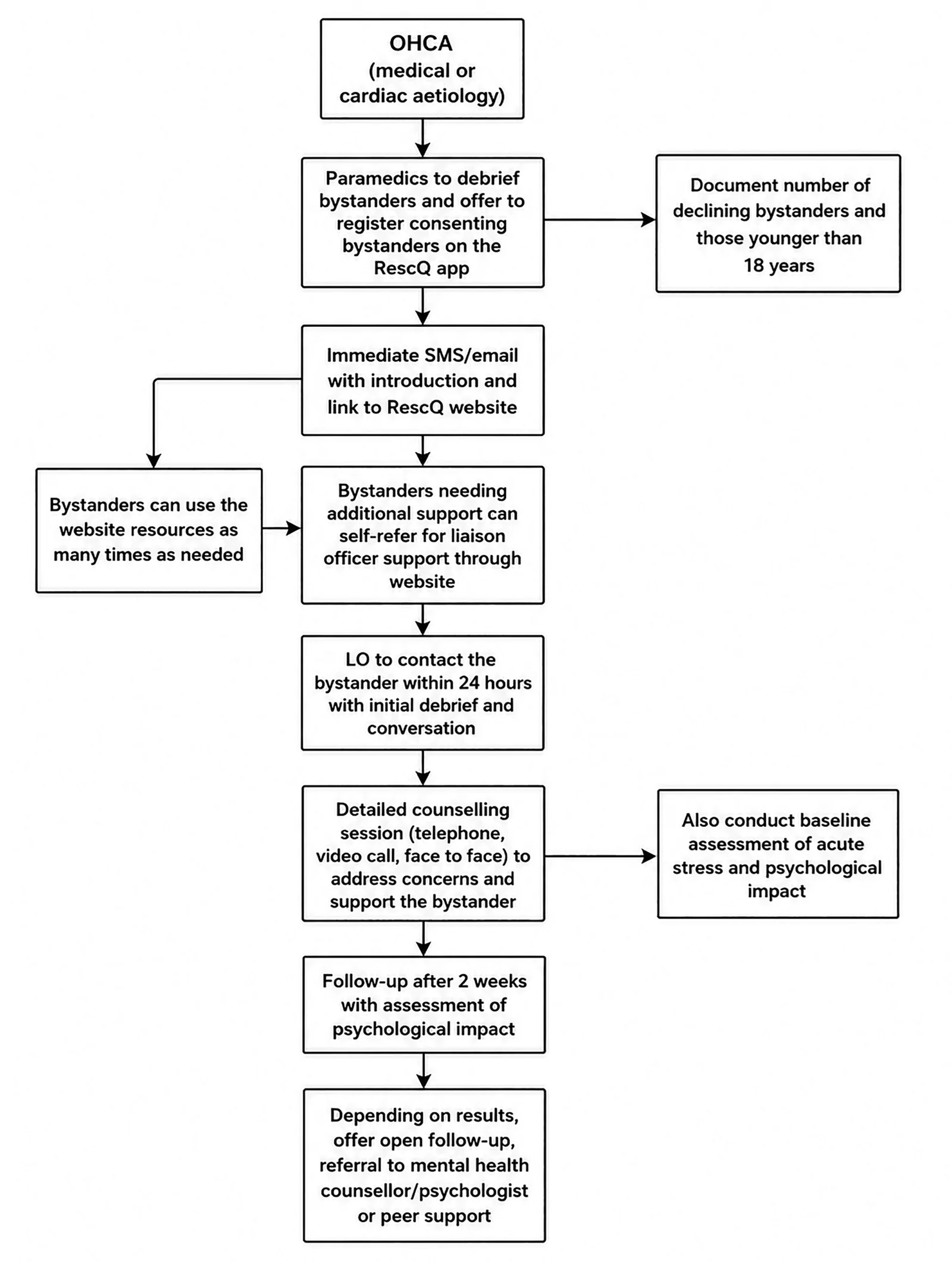
**Flow chart of inclusion and intervention**. Flow chart showing the inclusion pathway and intervention process for bystanders involved in out-of-hospital cardiac arrest of presumed medical or cardiac aetiology. Consenting bystanders are registered by ambulance clinicians at the scene and receive an immediate SMS/email link to the RescQ™ website. Bystanders may use the website resources independently and can self-refer for liaison officer support if additional support is required. Liaison officer contact includes initial supportive debriefing, baseline assessment of acute stress and psychological impact, follow-up assessment at two weeks, and onward signposting or referral where indicated. The number of declining bystanders and bystanders aged under 18 years is also recorded. **OHCA:** out-of-hospital cardiac arrest; **SMS:** short message service; **LO:** liaison officer.

**Fig. 3:**
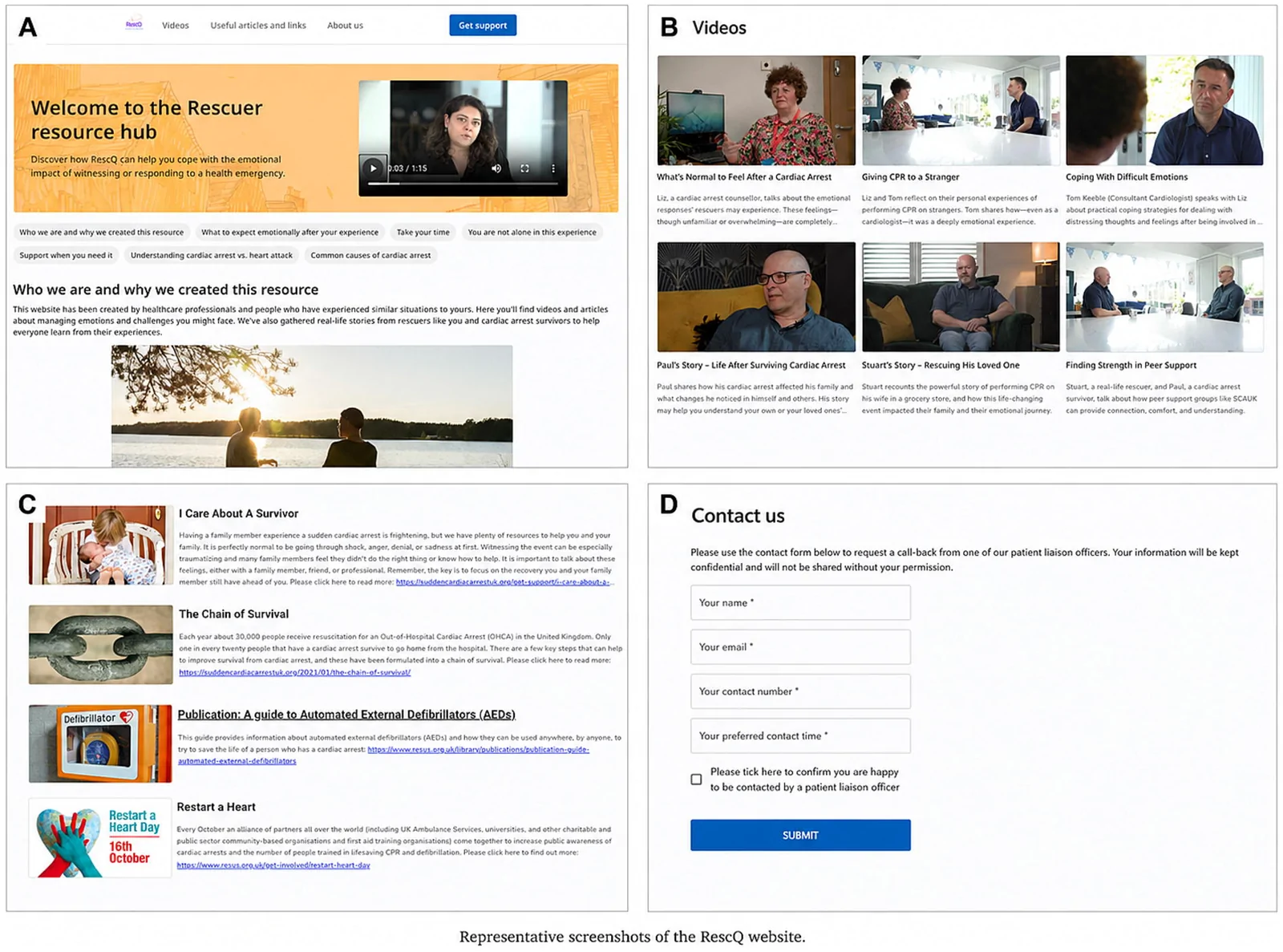
**Representative screenshots of the RescQ™ website**. (A) Homepage/resource hub introducing the purpose of the website and providing access to key sections. (B) Video resource library containing lived-experience and clinician-led videos addressing emotional responses, coping strategies, peer support and information about cardiac arrest. (C) Useful articles and external links section signposting bystanders to trusted cardiac arrest, cardiopulmonary resuscitation, defibrillation and support resources. (D) Self-referral form allowing bystanders to request contact from a liaison officer. The form requires the bystander to provide contact details and confirm consent to be contacted.

#### Liaison officer

Following self-referral, the LO makes an initial telephone contact to understand the bystander's needs and arranges a follow-up session, timed at least 72 h post-OHCA, in line with guidance on acute stress reactions.^21^, ^22^ Sessions are delivered by the bystander's preferred format, face-to-face, video call, or telephone, and may include a family member or friend.

At the start, bystanders complete a numerical stress rating^1^, ^2^, ^3^, ^4^, ^5^, ^6^, ^7^, ^8^, ^9^, ^10^ and validated screening tools, National Stressful Events Survey Short Scale (NSESSS)^23^ and Clinical Outcomes in Routine Evaluation-10 items (CORE-10)^24^ as baseline measures (Supplemental Appendix A). The session structure follows the Norwegian group follow-up model^19^: bystanders are invited to speak freely, with the LO providing acknowledgement, reassurance, and detailed responses to questions based solely on information shared, avoiding speculation about clinical decisions.

There is no fixed time duration for the LO session. A post-session stress rating is recorded, and bystanders are offered signposting, open follow-up, and referral to in-house psychological counselling or respective air ambulance LO services if further support is needed.

Where bystanders enquire about patient outcome, the LO responds with supportive discussion while maintaining confidentiality. Any disclosure of outcome information is managed according to local governance procedures and consent requirements.

#### Post LO intervention feedback survey

Bystanders receiving LO support will be invited to complete a structured feedback survey two weeks post-intervention, capturing website engagement patterns, psychological outcomes (NSESSS, CORE-10), and perceived usefulness of the website and LO support (rated 0–10). Free-text fields will also capture qualitative feedback and suggestions for improvement. The full bystander and ambulance clinician satisfaction surveys are provided in Supplemental Appendix B and C.

#### Consent process

The RescQ™ pathway is entirely consent driven. Ambulance clinicians provide a brief explanation of the pathway only when clinically and operationally appropriate, after patient care and scene safety have been prioritised. Bystanders may decline registration without giving a reason.

Website access is voluntary, and registered bystanders may use the resources at their own pace without engaging further.

Liaison officer support requires a separate voluntary self-referral through the website. Bystanders requesting LO contact provide consent for follow-up and may pause or stop contact at any time. They may also decline psychological screening, signposting or onward referral.

#### Ambulance clinician training

Relevant clinicians at EEAST have received structured education on the RescQ™ pathway, including training in use of the application and communication strategies for on-scene debriefing and introducing RescQ™ to bystanders.

#### Liaison officer training, supervision and role boundaries

The liaison officer role is not intended to provide formal psychotherapy or specialist mental health treatment. Within RescQ™, the liaison officer provides structured supportive contact, event-related discussion, psychological screening, reassurance, signposting and escalation where required.

The primary liaison officer is a senior cardiology trainee with clinical experience in managing critically unwell cardiac patients and communicating with patients and families after cardiac arrest. They have direct access to consultant cardiologists, a principal psychologist and mental health counselling support for advice, supervision and escalation.

Where severe distress, safeguarding concerns, risk of harm, or scores above predefined thresholds are identified, the liaison officer follows the RescQ™ escalation pathway. This may include signposting or referral to primary care, psychological services, mental health counselling, peer support or urgent crisis services, with consent where possible.

#### Data collection and follow-up (Table 2)

Baseline data will include OHCA incidents and bystanders registered. Website analytics will capture engagement metrics including page visits, time on resources, and video views. LO records will document bystander demographics, support provided, and onward referrals made.

**Table 2:** Data items collected during the study and the sources (OHCA: out-of-hospital cardiac arrest; LO: liaison officer, CORE-10: Clinical Outcomes in Routine Evaluation-10 items; NSESSS: National Stressful Events Survey Short Scale).

**Domain**	**Data item**	**Source**	**Time point**
OHCA event	Date/time, location/geolocation	RescQ™ app	At scene by ambulance clinicians
Bystander registration	Email address or cell phone number of the bystanders consenting for registration, known/unknown to patient, Number of bystanders declining registration and those under the age of 18 years	RescQ™ app	At scene by ambulance clinicians
Digital engagement	Unique users, sessions, page views, resource views, video views, time on resources	Website analytics	On accessing website
Liaison officer support	Timing of contact, mode of contact, duration, support provided, questions/concerns raised	LO record	At LO contact
Psychological measures	Self-stress score (1–10), CORE-10, NSESSS	LO assessment/follow-up survey	Baseline and two-week follow-up
Referral/signposting	Primary care, psychologist, mental health counsellor, peer support, air ambulance LO, other services	LO record	During/after LO contact
Acceptability	Website usefulness, LO usefulness, satisfaction ratings, free-text feedback	Bystander survey	Two weeks post-LO contact
Paramedic feedback	Awareness, usability, feasibility at scene, burden, acceptability, suggestions	Paramedic survey	During/after pilot
Service delivery	Staff role, registration time, LO time, admin time, supervision/training input, platform support	Study logs	Throughout pilot

Data from LO sessions and the two-week follow-up survey will undergo qualitative thematic analysis. Where stress scores exceed defined thresholds (NSESSS >14, CORE-10 >20), and with bystander consent, onward referral will be arranged to primary care, the ECTC psychologist, a mental health counsellor, or peer support networks such as SCAUK.

#### Safety monitoring and adverse event reporting

Although RescQ™ is a low-intensity supportive intervention, processes are in place to identify and respond to potential harm. Potential concerns include worsening distress, disclosure of self-harm or risk to others, safeguarding issues, complaints, inappropriate disclosure of confidential information, or need for urgent mental health or emergency support.

Any concerns identified during liaison officer contact are documented and escalated through the RescQ™ safety pathway. With consent where possible, bystanders may be signposted or referred to primary care, mental health services, crisis services or emergency services. Serious incidents or governance concerns are reported through local NHS incident reporting processes and reviewed by the RescQ™ clinical governance team.

### Outcomes

#### Primary outcome

The primary outcome is to determine the feasibility of delivering a structured bystander support programme following OHCA using the RescQ™ digital platform.

Feasibility will be assessed across three domains: (i) ambulance clinician uptake (registration of bystanders at the scene), (ii) bystander uptake (accessing RescQ™ website), and (iii) acceptability to both ambulance clinicians and bystanders.

Ambulance clinician uptake will be defined as the proportion of eligible OHCA incidents in which at least one bystander is registered via the RescQ™ app, with ≥40% considered indicative of feasibility.

Bystander uptake will be defined as the proportion of registered bystanders who access the RescQ™ website at least once, with the predefined threshold of ≥90% considered indicative of feasibility.

Acceptability will be assessed using a combination of uptake metrics, structured feedback and free-text responses. Ambulance clinician acceptability is assessed through a feedback questionnaire addressing awareness of RescQ™, ease of using the registration application, perceived suitability of introducing the pathway at scene, perceived burden on clinical workflow and suggestions for improvement (Supplemental Appendix C).

Bystander acceptability is assessed among those accessing liaison officer support using satisfaction ratings for the website and liaison officer intervention, perceived usefulness of support in coping with the event, willingness to recommend the service to others, and free-text feedback. Website access and self-referral rates are also interpreted as pragmatic indicators of acceptability and engagement.

**Fig. 4:**
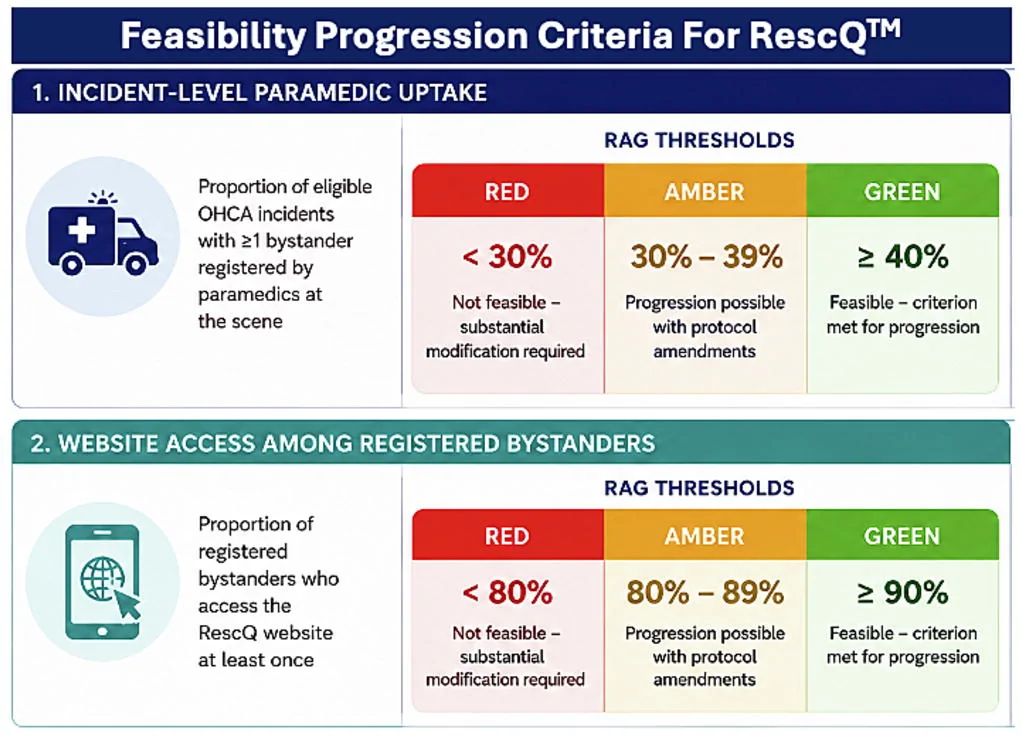
**RescQ™ pilot progression criteria for feasibility and acceptability**. Red-Amber–Green progression criteria for the two key feasibility outcomes in the RescQ™ pilot: incident-level ambulance clinician uptake and website access among registered bystanders. The thresholds were designed to assess feasibility and acceptability of the pathway rather than clinical effectiveness. **OHCA:** out-of-hospital cardiac arrest; **RAG** = red–amber–green. (For interpretation of the references to colour in this figure legend, the reader is referred to the web version of this article.)

Feasibility and acceptability outcomes will be interpreted using predefined thresholds, summarised in Fig. 4 and Table 3.

**Table 3:** RAG progression criteria for feasibility assessment (OHCA: out-of-hospital cardiac arrest).

**Feasibility domain**	**Green**	**Amber**	**Red**
Incident-level paramedic uptake: eligible OHCA incidents with ≥1 bystander registered	≥40%	30–39%	<30%
Website uptake: registered bystanders accessing website at least once	≥90%	80–89%	<80%

#### Secondary outcomes

1. Documentation of the number of bystanders <18 years
  • Enabling exploratory description of a group whose exposure to OHCA events is not currently known or captured by any reporting system.
2. Digital engagement and utilisation
  • Time from OHCA to first website access for the bystanders seeking LO support.
  • Frequency and depth of engagement (sessions, time spent, resources viewed).
  • Types of resources accessed (articles, videos, external links).
3. Psychological impact and support outcomes
  • Change in acute stress (1–10 scale) and validated scores (NSESSS, CORE-10) pre and 2 weeks post LO session.
  • Self-reported satisfaction with website and LO support.
  • Perceived usefulness of support in coping with the event.
4. Service-delivery and resource use outcomes
Service-delivery and resource utilisation data will be prospectively collected to assess operational and economic feasibility. Variables will include staff role and grade, time required for on-scene registration and debrief, LO intervention and admin time, supervision and training inputs, platform support, and onward referral activity.

### Subgroup comparisons

Engagement and utilisation will be explored by bystander relationship to the patient (known vs unknown) and age group (including under 18 s), where data are available. Exploratory comparisons will also be made between bystanders who access LO support and those who do not, limited to variables available across the full cohort.

Within the LO subgroup, further descriptive analyses will explore engagement patterns, timing of access, and baseline psychological measures. These analyses are hypothesis-generating in nature.

### Sample size and statistical analysis

As a feasibility pilot, RescQ™ is not powered to evaluate clinical effectiveness. However, two key binary progression criteria will be assessed using pre-specified red-amber-green thresholds: incident-level ambulance clinician uptake and website uptake among registered bystanders (Fig. 4).

For each criterion, the null hypothesis is that the true proportion falls within the red zone, with the design alternative set at the green-zone threshold. Exact one-sided binomial calculations will determine whether the anticipated denominator provides adequate power to reject this threshold. These calculations support feasibility interpretation and progression decisions, not clinical effectiveness testing.

For incident-level ambulance clinician uptake, green will be defined as ≥40% of eligible OHCA incidents having at least one bystander registered, amber as 30–39%, and red as <30%. A denominator of 193 eligible OHCA incidents would provide >90% power to reject a red-zone rate of ≤30%, assuming a true green-zone rate of ≥40%, using a one-sided exact binomial test with *α* = 0.05.

For website uptake, green will be defined as ≥90% of registered bystanders accessing the RescQ™ website at least once, amber as 80–89%, and red as <80%. A denominator of 112 registered bystanders would provide >90% power to reject a red-zone rate of ≤80%, assuming a true green-zone rate of ≥90%, using a one-sided exact binomial test with *α* = 0.05.

Observed proportions will be reported with exact binomial 95% confidence intervals (Clopper–Pearson method), interpreted alongside qualitative feedback, implementation context, and service-delivery data.

### Data sharing and dissemination

Anonymised and aggregate findings will be disseminated through peer-reviewed publications, conference presentations, patient and public involvement groups, ambulance service governance meetings and cardiac arrest support networks. Data sharing beyond the study team will be limited to anonymised aggregate outputs where disclosure risk has been assessed. The full dataset will not be made publicly available because it may contain sensitive information relating to bystanders, OHCA events and support needs. Full protocol is available in Supplementary Appendix D.

## Discussion

RescQ™ is a structured UK initiative to provide real-time psychological support for OHCA bystanders, integrating prehospital engagement, a tailored digital platform, and professional LO follow-up to address a critical gap in the chain of survival: the wellbeing of those who step forward to assist in resuscitation.

The RescQ™ design evolved considerably from initial conception. Early plans centred on QR code wristbands distributed at scene, but collaboration with ambulance services and SCAUK highlighted limitations around inconsistent uptake and lack of ongoing support. This led to a digital-first solution embedded within ambulance clinician workflow, enabling on-scene registration, immediate access to resources, and personalised follow-up, strengthening both feasibility and scalability.

RescQ™ is fully integrated into ambulance clinician workflow, enabling immediate bystander registration at scene. Its digital-first approach allows rapid delivery of resources, avoiding delays inherent in traditional referral pathways. The self-referral mechanism to LOs ensures personalised, flexible support, allowing bystanders to engage on their own terms and timeline.

Existing international bystander support programmes typically rely on helpline self-referral or volunteer responder app registration, leaving gaps for those who do not initiate contact or are unregistered. By embedding RescQ™ directly into the prehospital process, the programme ensures all identified bystanders are offered support, regardless of prior registration or proactive engagement.

Anticipated challenges include inconsistent ambulance clinician compliance given the high-pressure nature of OHCA scenes, variable digital literacy among bystanders, and declining engagement over time. Additionally, appropriate referral thresholds must be maintained, balancing accessible LO support with timely escalation to specialist mental health services where needed.

If proven feasible, RescQ™ has potential impact beyond OHCA, including traumatic and natural disasters. At a community level, improved bystander experience may increase confidence and willingness to intervene in future arrests. At a systems level, findings could inform NHS strategies for integrating psychosocial care into emergency response, with broader national and international relevance given the widespread absence of structured rescuer follow-up.

### Limitations

As a feasibility study, RescQ™ is not powered to demonstrate clinical efficacy in improving psychological outcomes. Data are self-reported and may be affected by response bias. Uptake may be influenced by digital access, language, or cultural barriers, potentially limiting generalisability. The self-referral model may underrepresent those most severely affected, who are least likely to engage digitally. Follow-up is currently limited to short-term outcomes; longer-term psychological trajectories remain unknown.

## Conclusion

While primarily a feasibility study, RescQ™ will provide early insights into the scale of unmet need and the potential role of structured support for bystanders following cardiac arrest.

## Current status of project

At the time of submission, the 12-month pilot has been completed, and data analysis is underway.

## Ethical approval

The study has been reviewed by West of Scotland regional ethical committee (REC Ref: 24/WS/0088, IRAS ID 340729) and was classified as a service evaluation, meaning formal NHS REC and HRA approval is not required. It is conducted in compliance with NHS (national health service) research governance and General Data Protection Regulation (GDPR). Information governance and data protection frameworks were established in collaboration with pre-hospital and hospital information teams, ensuring secure data handling and storage.

## Use of AI technology

Generative AI was used to assist with the visual preparation of Fig. 4 in this manuscript. The figure was generated based on author-provided content. The originality, accuracy, and appropriateness of the figure were reviewed and confirmed by the authors, who take full responsibility for the submitted content.

## Availability of data and other materials

No new data was generated in this study.

## CRediT authorship contribution statement

**Uzma Sajjad:** Writing – original draft, Visualization, Project administration, Methodology, Data curation, Conceptualization. **Marco Mion:** Writing – original draft, Visualization, Project administration, Methodology, Data curation, Conceptualization. **Rupert Simpson:** Writing – review & editing, Visualization, Methodology, Conceptualization. **Guilherme Movio:** Writing – review & editing, Project administration, Methodology, Data curation, Conceptualization. **Sarosh Khan:** Writing – review & editing, Visualization, Methodology, Conceptualization. **Liz Sharpe:** Writing – review & editing, Visualization, Project administration, Methodology, Conceptualization. **Jean Davis:** Writing – review & editing, Visualization, Project administration, Methodology, Conceptualization. **Luke Roberts-Andreou:** Writing – review & editing, Visualization, Project administration, Methodology, Conceptualization. **Gareth Grier:** Writing – review & editing, Visualization, Project administration, Methodology, Conceptualization. **Paul Swindell:** Writing – review & editing, Visualization, Project administration, Methodology, Conceptualization. **Stuart Menzies:** Writing – review & editing, Visualization, Supervision, Project administration, Methodology, Conceptualization. **Rob Major:** Writing – review & editing, Visualization, Project administration, Methodology, Conceptualization. **Blake Powell:** Writing – review & editing, Software, Project administration, Methodology, Conceptualization. **Michael Watts:** Writing – review & editing, Software, Project administration, Methodology, Conceptualization. **Nilesh Pareek:** Writing – review & editing, Visualization, Supervision, Methodology, Conceptualization. **Thomas R. Keeble:** Writing – review & editing, Visualization, Supervision, Resources, Project administration, Methodology, Funding acquisition, Conceptualization.

## Funding

Cardiac Network, East of England NHS.

## Declaration of competing interest

**SK** has received consulting and speaker fees from Vascular Perspectives and Astra Zeneca.

**NP** has received honoraria and educational grants from Abbott Vascular, and honoraria from NIPRO. NP serves on the Advisory Boards for Boston Scientific and Johnson & Johnson.

**TRK** has received grants from NHS East of England Cardiac Network and is unpaid chairperson for BCIS OHCA Focus group.

**US**, **MM**, **RS**, **GM**, **LS**, **JD**, **LRA**, **GG**, **PS**, **SM**, **RM**, **BP** and **MW** have no conflicts of interest.

## Appendix A Supplementary material

Supplementary Data 1 Templates for LO session structure, feedback surveys for ambulance clinicians and bystanders seeking LO support, and summary of the finalised RescQ protocol.
