# Supplementary material for: RescQ™: Study protocol for a feasibility pilot of structured support for bystanders/lay-rescuers following out-of-hospital cardiac arrest
Source: Resusc Plus. 2026 Aug 8;31:101445. doi: 10.1016/j.resplu.2026.101445 (PMC13506535; doi:10.1016/j.resplu.2026.101445)
Supplement: Supplementary file 1 [file mmc1.docx]

**Supplementary Appendix A**

RescQ^TM^ LO Intervention Template and Documentation Framework

*For use in the RescQ protocol manuscript*

| **Field** | **Details** |
| --- | --- |
| Project | RescQ^TM^ - support for bystanders involved in out-of-hospital cardiac arrest |
| Intervention | Liaison Officer (LO) supportive contact |
| Version | 1.3 |
| Date | 25/02/2025 |
| Prepared by | [Uzma Sajjad, Marco Mion, Liz Sharpe, Thomas R Keeble] |

# 1. PLO session structure

The order below provides the intended structure of the contact. In practice, the PLO may move between sections flexibly depending on the bystander response and needs.

| **Stage** | **Aim** | **Suggested wording / actions** |
| --- | --- | --- |
| 1. Introduction | Explain role and purpose of contact. | "My name is [name], and I am part of the RescQ team. I understand you were involved in a cardiac arrest event and requested some further support. This call is mainly to give you space to talk about what happened, answer questions where I can, and help identify whether any further support would be useful." |
| 2. Confirm willingness to proceed | Check privacy, safety and agreement to continue. | Confirm identity; check that the bystander is in a safe and private place; explain confidentiality and its limits; confirm they are happy to continue. |
| 3. Demographic and background details | Record brief baseline information without dominating the session. | Record age, gender, education, occupation, CPR training, previous CPR experience, relationship to patient and history of mental health issues where disclosed. |
| 4. Initial psychological assessment | Document baseline distress and identify current support needs. | Complete acute stress score and agreed baseline measures such as NSESSS, CORE-10 and HADS where used in the pathway. |
| 5. Bystander narrative | Allow the bystander to describe the event from their own perspective. | Prompts may include: "Can you talk me through what happened from your perspective?"; "What part has stayed with you the most?"; "Is there anything you would like to understand better?" |
| 6. Supportive debrief | Provide reassurance, clarification and normalisation where helpful. | Answer questions; explain general resuscitation processes; acknowledge emotional responses; highlight helpful actions taken by the bystander. Avoid pressing for details or giving inappropriate patient-specific information. |
| 7. Resource review | Explore use and usefulness of RescQ resources. | Ask whether the bystander accessed the website or videos, what was helpful or unhelpful, and whether any additional resources would be useful. |
| 8. Risk and safety-netting | Identify severe distress, functional impairment, safeguarding issues or risk. | Ask about coping, sleep, intrusive memories, avoidance, work or family function, support network and safety. Explore self-harm thoughts or inability to stay safe where clinically appropriate. |
| 9. Signposting / referral | Tailor support to need. | Offer RescQ resources, open appointment, planned follow-up, GP / NHS Talking Therapies / occupational health / counselling signposting, or urgent escalation according to local policy. |
| 10. Close and follow-up plan | Summarise, repeat safety-netting and agree next steps. | Repeat what has been discussed, check how the bystander feels after the call, complete post-session measures where feasible, and document follow-up or open appointment plan. |

# 2. Psychological measures

The LO records brief pre- and post-session measures where feasible. These measures are used to document current distress, guide immediate support needs and evaluate change around the PLO contact. They are not used as a stand-alone diagnostic assessment.

| **Measure** | **Timing** | **Purpose** |
| --- | --- | --- |
| Acute stress score | Pre-LO | Brief self-rated distress / stress level |
| NSESSS | Pre-LO | Assessment of acute stress-related symptoms where used in the pathway |
| CORE-10 | Pre-LO | Brief assessment of psychological distress |

# 3. Risk assessment and safety-netting

The LO explores whether the bystander is experiencing severe distress, functional impairment, safeguarding concerns or immediate risk. Any urgent concern is managed according to local escalation procedures.

| **Area assessed** | **Examples** |
| --- | --- |
| Current distress | Panic, intrusive memories, nightmares, avoidance, guilt, persistent tearfulness or agitation |
| Function | Ability to work, study, care for others, drive, sleep or complete usual activities |
| Support network | Family, friends, workplace, GP, existing mental health support or counselling |
| Risk | Thoughts of self-harm, feeling unsafe, inability to cope, safeguarding concerns or acute deterioration |

## Suggested safety question:

"Sometimes after an event like this, people can feel overwhelmed or unsafe. Have you had any thoughts of harming yourself, or felt that you might not be able to keep yourself safe?"

## Suggested safety-netting wording:

"If things feel worse, if you feel unsafe, or if you feel you are not coping, please seek urgent help through your GP, NHS 111, local crisis services or 999 in an emergency. You can also re-contact the RescQ team if you need further support."

# 4. Signposting and referral options

| **Need identified** | **Possible action** |
| --- | --- |
| Mild expected distress and good support network | Reassurance, RescQ resources, safety-netting and open appointment |
| Persistent distress or unanswered questions | Planned LO follow-up and further RescQ resources |
| Anxiety, low mood or functional impairment | GP review, NHS Talking Therapies / IAPT, occupational health or counselling services as appropriate |
| Severe distress, self-harm thoughts or immediate safety concern | Urgent escalation according to local policy, including crisis services, NHS 111 or 999 as appropriate |
| Interest in learning CPR or AED use | Signposting to CPR / AED training resources |
| Need for peer or community support | Signposting to appropriate survivor, bystander or cardiac arrest support organisations where available |

# 5. RescQ PLO documentation template

The following fields are recorded as part of the LO documentation framework. Local data collection forms may use the same variables in spreadsheet or electronic case report form format.

| **Domain** | **Variables** |
| --- | --- |
| Identification and dates | Initials / study identifier; incident date; request date; first contact date; LO session date |
| Registration and event details | Mode of RescQ registration; cause of OHCA; outcome of event |
| Session details | Duration of LO session; mode of LO session |
| Demographics | Age; gender; education; occupation |
| Bystander role | CPR trained; witnessed arrest; called 999; CPR done; previous real-time experience of CPR |
| Relationship to patient | Known to patient; relation; unknown to patient |
| Background factors | History of mental health issues where disclosed |
| Resource use | Used RescQ resources before PLO contact |
| Pre-session measures | Pre acute stress score; pre NSESSS; pre CORE-10 |
| Post-session measures | Post acute stress score; post NSESSS; post CORE-10 |
| Follow-up and escalation | Follow-up required; follow-up date; referral; signposting |

# 6. Session record form

This form can be copied into a local case report form or used as a session note template.

| **Section** | **Details to record** |
| --- | --- |
| Bystander identifier | [Initials / study ID] |
| Incident and registration | [Incident date; request date; registration mode] |
| Contact details | [First contact date; PLO session date; session mode; duration] |
| Bystander context | [Known/unknown to patient; relation; witnessed; called 999; CPR performed; CPR training] |
| Pre-session scores | [Acute stress score; NSESSS; CORE-10; HADS if used] |
| Main themes discussed | [Free-text summary of bystander concerns, questions, emotional response and key issues] |
| PLO support provided | [Reassurance; normalisation; debrief; questions answered; positive actions acknowledged; resources discussed] |
| Risk / safety concerns | [None identified / details / escalation action taken] |
| Signposting or referral | [Resources, GP, NHS Talking Therapies, occupational health, counselling, crisis pathway, other] |
| Post-session scores | [Acute stress score; NSESSS; CORE-10; HADS if used] |
| Follow-up plan | [No further contact / open appointment / planned follow-up date / referral] |

# 7. Intervention fidelity checklist

| **Core component** | **Completed** |
| --- | --- |
| PLO introduced role and purpose of contact | [ ] |
| Bystander identity, privacy and willingness to proceed confirmed | [ ] |
| Confidentiality and limits explained | [ ] |
| Basic demographic and background details recorded where available | [ ] |
| Pre-session psychological measures completed where feasible | [ ] |
| Bystander given opportunity to describe the event in their own words | [ ] |
| Supportive listening, validation and normalisation provided | [ ] |
| Questions answered or debrief provided where requested and appropriate | [ ] |
| Helpful actions taken by the bystander acknowledged where relevant | [ ] |
| Use of RescQ resources reviewed | [ ] |
| Risk and safety concerns explored | [ ] |
| Safety-netting advice provided | [ ] |
| Signposting or referral documented | [ ] |
| Post-session psychological measures completed where feasible | [ ] |
| Follow-up plan or open appointment documented | [ ] |

**Supplementary Appendix B**

**RescQ^TM^ Bystander Follow-up Survey**

*Survey sent approximately two weeks after Patient Liaison Officer (PLO) intervention*

| **Survey title** | RescQ User Satisfaction Survey |
| --- | --- |
| **Time point** | Approximately two weeks after LO support/contact |
| **Target respondent** | Bystander or lay-rescuer registered through the RescQ pathway, who have sought PLO support |
| **Estimated completion time** | 5–7 minutes |
| **Purpose** | To evaluate website use, perceived helpfulness of LO support, current wellbeing, signposting/referral needs, and suggestions for improving support for future bystanders. |
| **Confidentiality statement** | All responses remain confidential. |
| **Source format** | Online Tally survey converted into a Word template for manuscript supplementary material. |

# Participant-facing introduction

**Thanks for using RescQ – Support for the Bystander.**
We would really value your feedback. It only takes 5–7 minutes, but what you share will help us improve RescQ and make sure other bystanders get the best support possible. Your voice truly makes a difference. All responses will stay confidential.

## A. Respondent identifier

| **No.** | **Question** | **Response format / options** | **Required** |
| --- | --- | --- | --- |
| **1** | What is your preferred name or initials? | Short free-text response | Yes |

## B. RescQ website use and feedback

| **No.** | **Question** | **Response format / options** | **Required** |
| --- | --- | --- | --- |
| **2** | When did you first visit the RescQ website after receiving the link? | A. Within a day  B. Within the first few days  C. Within a week  D. Within a month  E. Within two months | Yes |
| **3** | How easy did you find using the website? | 0–10 rating scale | Yes |
| **4** | How helpful or relevant did you find the information on the website? | 0–10 rating scale | Yes |
| **5** | How many times have you used the website since? | A. Once  B. Twice  C. A few times  D. Lots of times | Yes |
| **6** | What did you find helpful on the website? Please include any specific article or video if relevant. | Free-text response | No |
| **7** | What did you find the least helpful on the website? | Free-text response | No |
| **8** | Is there anything you would like to see on the website that is not there? | Free-text response | No |

## C. PLO support feedback

| **No.** | **Question** | **Response format / options** | **Required** |
| --- | --- | --- | --- |
| **9** | How easy was it to request support from the Patient Liaison Officer (PLO)? | A. Easy  B. Difficult, as I could not find the tab/button for it  C. Difficult, because of personal/emotional reasons | Yes |
| **10** | How helpful was the conversation or support you received from the PLO? | 0–10 rating scale | Yes |
| **11** | How did the PLO support help you? | Select all that apply, if relevant:  A. Answered my questions about CPR  B. Made me feel better about my involvement in the situation  C. Answered questions about the possible outcomes  D. Provided emotional support  E. Signposted to other resources | No |
| **12** | Was there anything that stood out, positively or negatively, about the PLO contact? | Free-text response | No |

## D. Current subjective wellbeing

| **No.** | **Question** | **Response format / options** | **Required** |
| --- | --- | --- | --- |
| **13** | How do you feel now? | A. I feel okay about the event  B. I feel more emotionally or psychologically overwhelmed since the event  C. I feel the same as I did before the PLO session  D. I feel less emotionally or psychologically overwhelmed since the event | Yes |

## E. Acute stress symptom items

How much have you been bothered during the past seven (7) days by each of the following problems?

| **No.** | **Statement** | **Not at all** | **A little bit** | **Moderate** | **Quite a bit** | **Extremely** |
| --- | --- | --- | --- | --- | --- | --- |
| **1** | Having flashbacks; that is, you suddenly acted or felt as if a stressful experience from the past was happening all over again, for example, you re-experienced parts of a stressful experience by seeing, hearing, smelling, or physically feeling parts of the experience. | ☐ | ☐ | ☐ | ☐ | ☐ |
| **2** | Feeling very emotionally upset when something reminded you of a stressful experience. | ☐ | ☐ | ☐ | ☐ | ☐ |
| **3** | Feeling detached or distant from yourself, your body, your physical surroundings, or your memories. | ☐ | ☐ | ☐ | ☐ | ☐ |
| **4** | Trying to avoid thoughts, feelings, or physical sensations that reminded you of a stressful experience. | ☐ | ☐ | ☐ | ☐ | ☐ |
| **5** | Being super alert, on guard, or constantly on the lookout for danger. | ☐ | ☐ | ☐ | ☐ | ☐ |
| **6** | Feeling jumpy or easily startled when you hear an unexpected noise. | ☐ | ☐ | ☐ | ☐ | ☐ |
| **7** | Being extremely irritable or angry to the point where you yelled at other people, got into fights, or destroyed things. | ☐ | ☐ | ☐ | ☐ | ☐ |

Required: Yes

## F. CORE-10 wellbeing items

The following 10 statements are about how you have been over the last week. Please read each statement and think how often you felt that way last week. Then tick the box that is closest to this.

| **No.** | **Statement** | **Not at all** | **Only occasionally** | **Sometimes** | **Often** | **Most of the time** |
| --- | --- | --- | --- | --- | --- | --- |
| **1** | I have felt tense, anxious or nervous. | ☐ | ☐ | ☐ | ☐ | ☐ |
| **2** | I have felt I have someone to turn to for support when needed. | ☐ | ☐ | ☐ | ☐ | ☐ |
| **3** | I have felt able to cope when things go wrong. | ☐ | ☐ | ☐ | ☐ | ☐ |
| **4** | Talking to people has felt too much for me. | ☐ | ☐ | ☐ | ☐ | ☐ |
| **5** | I have felt panic or terror. | ☐ | ☐ | ☐ | ☐ | ☐ |
| **6** | I made plans to end my life. | ☐ | ☐ | ☐ | ☐ | ☐ |
| **7** | I have had difficulty getting to sleep or staying asleep. | ☐ | ☐ | ☐ | ☐ | ☐ |
| **8** | I have felt despairing or hopeless. | ☐ | ☐ | ☐ | ☐ | ☐ |
| **9** | I have felt unhappy. | ☐ | ☐ | ☐ | ☐ | ☐ |
| **10** | Unwanted images or memories have been distressing me. | ☐ | ☐ | ☐ | ☐ | ☐ |

Required: Yes

## G. Further support and final comments

| **No.** | **Question** | **Response format / options** | **Required** |
| --- | --- | --- | --- |
| **14** | Have you spoken to a GP, counsellor, or mental health professional about any issues regarding the event since the PLO support? | A. Yes  B. No  C. I plan to | Yes |
| **15** | Is there anything else you would like to share about your experience, or how we could better support bystanders in future? | Free-text response | Yes |

# Optional data export fields

The following field categories may be used when exporting or analysing survey responses: respondent identifier, website access timing, website ease-of-use rating, website relevance/helpfulness rating, website usage frequency, qualitative website feedback, ease of LO support request, LO helpfulness rating, perceived mechanism of LO support, current subjective wellbeing, acute stress symptom responses, CORE-10 responses, onward help-seeking, and final free-text comments.

Supplementary Appendix C
Prehospital Clinician Satisfaction Survey

*RescQ^TM^ clinician feedback survey*

This appendix reproduces the clinician feedback survey used to evaluate the RescQ app among EEAST clinicians. The survey captures clinician role, cardiac arrest exposure, app use, usability, technical reliability, perceived impact on scene communication/debriefing, and suggestions for improvement.

# Survey introduction

Thanks for taking the time to use RescQ to support the bystanders. To help us improve RescQ for both EEAST clinicians and the bystanders, we'd like to ask you a few questions about your experience so far. It'll only take 5 minutes and your answers will help us make RescQ better for you.

# Survey items

### 1. What title best describes your role in EEAST? *

**Response format:** Single choice

☐ Paramedic / Nurse

☐ Leading Operations Manager

☐ Emergency Medical Technician

☐ HART Paramedic

☐ HEMS Paramedic

☐ Community First Responder

### 2. Which of the following is your base station? *

**Response format:** Single choice

☐ Chelmsford

☐ Southend

☐ Basildon

☐ Harlow

☐ Colchester

☐ Other

### 3. Roughly how many cardiac arrest cases have you attended in the past 3 months? *

**Response format:** Single choice

☐ None

☐ 1-5

☐ 6-15

☐ 16 or more

### 4. Have you used the app in a real patient encounter? *

**Response format:** Single choice

☐ Yes

☐ No

### 5. How often do you use the app in cardiac arrest cases? *

**Response format:** Single choice

☐ Every time

☐ Most of the time

☐ Half of the time

☐ A quarter of the time

☐ Never

### 6. How easy is the app to navigate and use?

**Response format:** Rating scale

☐ 0

☐ 1

☐ 2

☐ 3

☐ 4

☐ 5

☐ 6

☐ 7

☐ 8

☐ 9

☐ 10

### 7. Did the app load quickly and work reliably? *

**Response format:** Single choice

☐ Always

☐ Most of the time

☐ Sometimes

☐ Rarely

☐ Never

### 8. Were any technical issues or bugs encountered? *

**Response format:** Free text

_____________________________________________________________________________________

_____________________________________________________________________________________

_____________________________________________________________________________________

### 9. How do you use the app to register the bystanders? *

**Response format:** Single choice

☐ I prefer registering the bystanders myself, to ensure access to resources

☐ I prefer to give out the QR code for the bystanders to scan and use themselves

☐ No preference, depends on the occasion

### 10. Do you feel the app improved any of the following for you? *

**Response format:** Select all that apply

☐ Ensuring an on-scene debrief is given to the bystanders

☐ Confidence that the bystanders will be supported

☐ Communication with the bystanders at the scene

☐ Reducing your own emotional burden after difficult cases

☐ Time efficiency in providing debrief

☐ Made the debrief on scene longer

☐ Made no difference to the communication with the bystanders at the scene

☐ Has not helped me navigate the situation with bystanders on the scene

### 11. On a scale of 0-10, how helpful has this app been to your clinical practice so far? *

**Response format:** Rating scale

☐ 0

☐ 1

☐ 2

☐ 3

☐ 4

☐ 5

☐ 6

☐ 7

☐ 8

☐ 9

☐ 10

### 12. In the last month, have you come across colleagues who did not know about this app? *

**Response format:** Single choice

☐ Yes

☐ No

### 13. Anything else you'd like us to know? Any suggestions/feedback

**Response format:** Free text

_____________________________________________________________________________________

_____________________________________________________________________________________

_____________________________________________________________________________________

### 14. Any reason why you have not been using the app? *

**Response format:** Free text

**Note:** This item may be used as a follow-up or conditional question for respondents who report not using the app.

_____________________________________________________________________________________

_____________________________________________________________________________________

_____________________________________________________________________________________

**Appendix D**

**RescQ™ feasibility pilot protocol summary**

**Title**

**RescQ™: a feasibility pilot of a structured support pathway for bystanders involved in out-of-hospital cardiac arrest**

**Protocol version**

Protocol version: v1.6

Protocol date: 01/02/2025

**Purpose Creating support for co-survivors/rescuers**

The scene of a cardiac arrest is a busy place, paramedics and other pre-hospital teams (air ambulance crews etc) have invested an incredible effort in resuscitating a patient and stabilizing them for transfer. Time is of the essence to get the patient definitive care and pre-hospital teams are busier than ever; as a result, there is often insufficient time for an on scene debrief, check of co-survivor welfare or sign posting to approved literature and peer-to-peer support.

**Study design**

RescQ™ is a 12-month, non-randomised, prospective observational feasibility pilot evaluating a structured support pathway for adult bystanders involved in out-of-hospital cardiac arrest of presumed cardiac or medical aetiology. The study is designed to assess feasibility, acceptability, digital engagement and preliminary psychological outcomes, rather than clinical effectiveness.

**Collaborators**

1. Essex Cardiothoracic Centre, Mid and South Essex NHS Trust
2. Anglia Ruskin University, Chelmsford
3. EEAST (East of England Ambulance Service NHS Trust)
4. EHAAT (Essex and Herts Air Ambulance NHS Trust)
5. EAAA (East Anglian Air Ambulance)
6. SCAUK (Sudden Cardiac Arrest UK)

**Pilot Project Plan**

The aim of this project is twofold:

**1.** To provide co-survivors with on the scene signposting to a respected source (website) containing information for their own reading

**2.** Give them the opportunity to self-refer to patient liaison officers for an opportunity to provide:

**a.** further feedback on the resuscitation - we know that co-survivors often need the confirmation that they did a good job / followed dispatcher advice / did their best regardless of the outcome.

**b.** Onward signposting for ongoing issues outside the remit of the patient liaison officer, including referral to primary care / Mental Health services, counselling accordingly.

**Setting**

The pilot is conducted across Essex, United Kingdom, within the regional out-of-hospital cardiac arrest pathway involving East of England Ambulance Service NHS Trust, Essex and Herts Air Ambulance Trust, East Anglian Air Ambulance, local emergency departments and Essex Cardiothoracic Centre.

**Participants**

Eligible participants are adults aged 18 years or older who witnessed, called 999 for, or directly assisted in resuscitation during an out-of-hospital cardiac arrest of presumed cardiac or medical aetiology.

Bystanders are excluded if they are under 18 years of age, unable to provide consent, too distressed to engage at the scene, unable to provide contact details, or involved in cardiac arrests due to trauma, road traffic accident, drug overdose, suicide or drowning.

Bystanders under 18 years of age are not registered into the intervention, but their presence at eligible events is recorded descriptively where possible.

**Intervention pathway**

The RescQ™ pathway has three main stages.

First, ambulance clinicians provide a brief explanation of the pathway and offer registration when clinically and operationally appropriate, after patient care and scene safety have been prioritised. Registration is completed using the RescQ™ application on ambulance service devices.

Following registration, the bystander’s contact details are transferred to a secure central server and automatically deleted from the clinician-facing application.

Second, registered bystanders receive a single text message or email containing a secure link to the RescQ™ website. The website provides information on common physical and psychological reactions after witnessing cardiac arrest or performing cardiopulmonary resuscitation, coping strategies, lived-experience videos, clinician-led information and signposting to further support.

Third, bystanders who require additional support can voluntarily self-refer through the website to request liaison officer contact. Liaison officer support includes structured supportive contact, event-related discussion, psychological screening, reassurance, signposting and onward referral where required.

**Consent process**

The pathway is entirely consent driven. Bystanders may decline registration without giving a reason. Website access is voluntary, and registered bystanders are not required to complete questionnaires or engage further.

Liaison officer support requires a separate voluntary self-referral through the website. Bystanders requesting liaison officer contact provide consent for follow-up and may pause or stop contact at any time. They may also decline psychological screening, signposting or onward referral.

**Liaison officer role and supervision**

The liaison officer role is not intended to provide formal psychotherapy or specialist mental health treatment. The primary liaison officer is a cardiology trainee with 13 years of clinical experience managing out-of-hospital cardiac arrest, critically unwell cardiac patients and communication with patients and families after cardiac arrest.

The liaison officer is trained in the RescQ™ pathway, trauma-informed communication, confidentiality, safeguarding, use of CORE-10 and the National Stressful Events Survey Short Scale, recognition of severe distress and referral pathways. They are directly supervised by consultant cardiologists and have access to psychology and mental health counselling input for advice and escalation.

**Outcomes**

The primary outcome is feasibility of delivering the RescQ™ pathway.

Feasibility is assessed through:

| **Feasibility domain** | **Green** | **Amber** | **Red** |
| --- | --- | --- | --- |
| Eligible OHCA incidents with at least one bystander registered | ≥40% | 30–39% | <30% |
| Registered bystanders accessing the RescQ™ website at least once | ≥90% | 80–89% | <80% |

Secondary outcomes include digital engagement, liaison officer self-referral, timing and mode of liaison officer contact, psychological screening scores, onward referral activity, bystander satisfaction, ambulance clinician feedback and service-delivery/resource-use data.

**Data collection**

Data are collected from the RescQ™ application, website analytics, liaison officer documentation, bystander follow-up questionnaires, ambulance clinician feedback questionnaires and study logs.

Collected data include event registration, number of bystanders registered, declined registrations, bystanders under 18 years of age, website access, resource use, video views, liaison officer contact details, self-reported stress score, CORE-10, National Stressful Events Survey Short Scale, referral activity, satisfaction ratings and free-text feedback.

| **Domain** | **Data item** | **Source** | **Time point** |
| --- | --- | --- | --- |
| OHCA event | Date/time, location/geolocation | RescQ app | At scene by ambulance clinicians |
| Bystander registration | Email address or cell phone number of the bystanders consenting for registration, known/unknown to patient,  Number of bystanders declining registration and those under the age of 18 years | RescQ app | At scene by ambulance clinicians |
| Digital engagement | Unique users, sessions, page views, resource views, video views, time on resources | Website analytics | On accessing website |
| Liaison officer support | Timing of contact, mode of contact, duration, support provided, questions/concerns raised | Liaison officer record | At LO contact |
| Psychological measures | Self-stress score (1–10), CORE-10, National Stressful Events Survey Short Scale | LO assessment / follow-up survey | Baseline and two-week follow-up |
| Referral/signposting | Primary care, psychologist, mental health counsellor, peer support, air ambulance LO, other services | LO record | During/after LO contact |
| Acceptability | Website usefulness, LO usefulness, satisfaction ratings, free-text feedback | Bystander survey | Two weeks post-LO contact |
| Paramedic feedback | Awareness, usability, feasibility at scene, burden, acceptability, suggestions | Paramedic survey | During / after pilot |
| Service delivery | Staff role, registration time, LO time, admin time, supervision/training input, platform support | Study logs | Throughout pilot |

**Safety and escalation**

Potential concerns include worsening distress, disclosure of self-harm or risk to others, safeguarding concerns, complaints, inappropriate disclosure of confidential information, or need for urgent escalation. Any concerns identified during liaison officer contact are documented and escalated through the RescQ™ safety pathway. This may include signposting or referral to primary care, psychological services, mental health counselling, peer support, crisis services or emergency services as appropriate.

**Governance and data protection**

The study was reviewed by the West of Scotland Regional Ethics Committee and classified as a service evaluation. Formal NHS Research Ethics Committee and Health Research Authority approval were not required. The pilot is conducted in accordance with NHS governance requirements and the General Data Protection Regulation.

Identifiable data are stored securely and accessed only by authorised members of the RescQ™ team. Findings will be reported using anonymised or aggregate data.

Data sharing agreements and DPIA have been contracted between Essex cardiothoracic Centre, EEAST, EHAAT, EAAA and Blum Health Ltd.

**Dissemination**

Findings will be disseminated through peer-reviewed publication, conference presentation, NHS and ambulance service governance meetings, patient and public involvement groups, cardiac arrest support networks and lay summaries for survivors, co-survivors and bystanders.

**Collaborator Faculty**

Dr Uzma Sajjad ^1^

Dr Thomas Keeble ^2^

Dr Gareth Grier ^4^

Dr Rob Major ^5^

Luke Roberts-Andreou^6^

^1^ Cardiology Department, Essex Cardiothoracic Centre, Basildon and Thurrock University Hospitals NHS Trust, U.K.

^2^ Anglia Ruskin University, Chelmsford, U.K, Essex Cardiothoracic Centre, Basildon Hospital NHS Trust, RCUK and BCIS OHCA Focus Group Chair

^3^ Essex and Herts Air Ambulance NHS Trust, UK

^4^ East Anglian Air Ambulance

^5^ East of England Ambulance Services
